# American Academy of Dental Science

**Published:** 1902-09

**Authors:** 

**Affiliations:** American Academy of Dental Science


### Reports of Society Meetings.

### AMERICAN ACADEMY OF DENTAL SCIENCE.

THE regular monthly meeting of the American Academy of Dental Science was held at Young's Hotel, Boston, on Wednesday evening, March 5, 1902, at six o'clock, President Bradley in the chair.

### INCIDENTS OF PRACTICE.

*President Bradley.*—Under the head of "Incidents of Office Practice," I believe Dr. Cecil P. Wilson has something of interest which he will present to the Academy.

*Dr. Wilson.*—I have a mallet, the model of which I got up some little time ago. It works on a different principle from the ordinary automatic mallet. This mallet has an outer case just like any other mallet. The inner plunger or shaft connects with this right-angle piece, and that, in turn, connects with what is called a rachet gear, and that operates a small pinion-wheel gear, which forces the mallet down. The length of the full stroke is about one-tenth of an inch; that brings the hammer down ten-tenths of an inch, or ten to one. Inside of the mallet is a spring, very similar to any other mallet, simply for the purpose of returning, or bringing back, the hammer-head to its previous position after the blow has been struck. There is also a small spring connected with the hammer-arm, for the same purpose. The hammer-head is a piece of brass, with a hole bored directly through the centre. The lower end of this hole is stopped with a piece of thin white metal, and over this piece of white metal lead is pounded down. The plunger coming in contact with the white metal, with a backing of lead, gives an effective blow, and at the same time one that is not disagreeable.

*Dr. Andrews.*—I had the pleasure of seeing this mallet work in Dr. Wilson's office, not very long ago, and I was very much pleased with the way it packed gold. Any one who will try it will find it works differently from the ordinary hand-mallet. I was so much pleased with it that I ordered one.

*Dr. Smith.*—Dr. Wilson kindly presented me with one of his mallets, and I am very much pleased with it. The action is somewhat different from the ordinary automatic mallet, and it comes the nearest to the old-fashioned hand-mallet of any automatic mallet I have ever seen.

*Dr. Wilson.*—In regard to the hammer-head, it is a piece of brass with a hole bored directly through the centre, and the plunger comes in contact with a piece of platinum. This platinum is soldered directly in the bottom of the hammer, and then the hammer is turned over and lead is pounded down into it. If it were poured in, it would be too soft, but it is hammered down hard. I think the platinum is just a grain soft. This only comes in contact with a piece of white metal instead of platinum. I forgot to explain that.

*President Bradley.*—Dr. Brackett has an appliance that he would like to present.

*Dr. Brackett.*—There came to me at the beginning of the summer season last year in Newport a gentleman from another city, in a very unsatisfactory condition, on account of difficulties with an upper plate. He had had several plates made by a number of different dentists, and none of them with any success whatever, so far as suction or stability was concerned. The patient was a gentleman of refinement and of social instincts, and his condition very much impressed people who had not seen him since the loss of the last of his natural upper teeth. With his upper plate in his mouth he could speak little except by closing his lower teeth against the others and talking through his teeth, which was a very serious embarrassment for him.

I do not remember another instance where trouble of this sort with artificial teeth was so grievous as in the case of this man. The other members of the club which he frequented, quite a number of them, at different times came to me with an appeal: "Is it not posible to do something for Mr. So-and-so's teeth?" and he was denying himself those social pleasures to which he was accustomed. It was a matter of remark in the community, "Why is it that Mr. So-and-so accepts no dinner invitations this year?" His condition in this particular appealed very much to my sympathies. I have a great esteem for this gentleman, and I was very much impressed with the seriousness of the predicament in which he was. I said that I did not know that I could do anything for him, but I would try. I did try, from early in the summer until the day before his return to his city home in the autumn, when he came away from Newport with the appliance which I show you to-night.

The peculiar difficulty was with his upper arch, which was very small and extremely soft and pliable. I pass about two models, one of which shows the cast just as it came from the impression, and another one which happened to be by its side in the laboratory, and upon which I did considerable carving about the borders, hoping that turning in the border of the plate would help. If I compare the mouth to anything I ever saw, it would be perhaps to a uvula in texture and mobility. I made a number of patterns, including some of the Ideal base-plate, which is inflexible, and, trying them, I was able to make nothing whatever in the way of a pattern of any material that would stay in place for an instant. I settled upon the conviction that the plate, which had been made for him by a capable practitioner in Boston, was probably the best that could be done. Then I began to think what manner of scheme could be contrived by which that upper plate could be held up. He wore on the left side of his lower jaw a partial set of a few teeth, not filling all of the spaces, and he had a number of natural teeth remaining standing at various angles, the left lower second bicuspid in particular being very much tipped inward. I conceived the idea of using spiral springs, after the old-fashioned English plan, to hold up the upper plate; and, taking the upper plate with which the patient was already provided, I began making efforts at the construction of a lower plate which I could put in the mouth after it was made. I tried a good many times before succeeding. The way the lower plate was ultimately constructed was by making one section at a time, filling one space with a new construction of plate, putting that in place, taking an impression of another section and completing that, and then the third section likewise, in this way making one continuous partial lower plate with three vulcanizations.

All of this was so experimental that I did not feel justified in taking away anything whatever from the lower jaw which was serviceable as a support for the appliance which he was already wearing. If I failed, I wished to leave him no worse off than I found him. This lower plate, being constructed in sections in that way, had attached to it. after the manner which you will see, a spiral spring on each side turning on a swivel, with clips upon the lower molars, so that the force of that spring should not drive the lower plate too much down into the soft tissues. It was immediately successful, and has been worn from October to the present time with comfort and satisfaction. The lower plate I made was so thin on the lower jaw, in order to spring it into place, that it broke, but it was readily repaired, as you see. The patient puts in this fixture in the morning and wears it down town to his office and attends to his business. As he is quietly in his home in the afternoon, he sometimes finds the presence of the springs a little uncomfortable; when that is experienced he takes out the plate and gives the mouth a rest. The springs suffice to hold up the plate. The appliance enables the gentleman to attend an evening dinner if he chooses, partake of food, and open his mouth to speak without embarrassment or difficulty.

Of the few humble accomplishments within the limits of my practice of dentistry, there are few that have given me greater pleasure than what I have been enabled to do for this gentleman. When I asked him a week ago if he would permit me to bring this appliance here to show to you, he answered, "By all means; if this can be of any service to anybody else, I will gladly have it exhibited;" so it has been taken from his mouth to-night and brought here for you to see. I would say that the credit of this admirably fitted upper plate belongs to Dr. W. B. Parker, of this city.

Dr. Parker has most kindly written me: "I was very glad when Mr. —— told me that you had succeeded in making his plate stay up. He was very patient with my experiments last winter, and I was sorry for him. I tried every way I could think of except the springs, which of course I ought to have tried, but remembering the old times when springs were used so much, and the trouble they gave, I thought the chance of success pretty slight. I think the subject of to-morrow evening may be an interesting one, and am sorry that I cannot be there."

I take the liberty of saying further that the patient, at the instance of his physician, gave one of our members an opportunity to see his mouth, and to make one effort at the construction of something on the upper jaw. The patient did this in perhaps a rather perfunctory way, and did not give the dentist, who may perhaps testify something about the condition of the mouth, the opportunity which he desired to persevere with it. I would say further that I have used the springs in other cases where there was a necessity for some contrivance for holding up the upper plate. One was the case of a gentleman who had lost every vestige of his soft palate and all of his hard palate except a rim around the alveolar process, and had also lost all of his nose, so that his face was perfectly flat. So long as he had some natural teeth remaining on his upper jaw I was able to hold up the plate and an artificial nose in place with a spiral spring, the back end of which was attached to a staple in the upper surface of the plate and the forward end to the back of the septum of his artificial nose. When all the natural teeth were gone, spiral springs, resembling these, became an indispensable and efficient resource, and they have been so worn many years.

The problem in both of these cases was to give a full upper plate stability under circumstances of peculiar difficulty. These springs have provided the solution. In the case of the patient without palate or nose, trial was made of the Stedman springs, but they were quite inefficient. The use of springs is most frequent, I think, for entire dentures where there are no teeth whatever on the lower jaw, and generally with the object, largely, of giving the lower plate stability. Their use in a case resembling this which I show is, I think, unusual.

*President Bradley.*—Any remarks to be made on this pattern?

*Dr. Allen.*—A short time before this patient came to Dr. Brackett, I had the pleasure of seeing his case, and of trying to relieve his trouble. I found that the plate he was wearing, the one which you see now, was a very perfect fit, so far as it was possible to adapt a plate to these peculiar conditions, but I was requested to see if I could make a plate which would have some suction. I saw the difficulties of the case, and doubted whether I could improve upon the plate that he was then wearing; nevertheless, I made the attempt. I do not know how my efforts would have succeeded, because when the plate that I constructed came back from the laboratory workman it was so porous as to be useless, and as the patient was soon to leave the city, I was unable to give the case further attention. I do not believe, however, that I should have succeeded. I think the same difficulty would have been encountered with the plate that I had attempted as with the other. It did not occur to me to try the springs at that time, as I have inherited the prejudice against them that is so prevalent in the profession to-day. But I congratulate Dr. Brackett upon his success in solving a difficult problem, and feel that I have learned something thereby.

*Dr. Fillebrown.*—I think perhaps the matter of springs will be considered more by the profession in the future for these cases than in the past. We discard a thing, and then later rediscover it and take it up again.

It was twenty years ago or more that I had occasion to use springs, but not to hold up an upper plate, but to hold down an under plate. I had a patient for whom I made a full set of teeth. Her under jaw was very short, her chin receding, and whenever she opened her mouth she squeezed her under teeth right out. They would not stay in at all. I remedied the defect by putting on a set of springs, and she wore them with great comfort so long as I knew her. In looking at this case as it passed me, it strikes me that the springs are a good deal stiffer than is necessary, and I think the gentleman can have a pair of springs put on that plate that are so gentle in their action that he will wear them the twenty-four hours without excessive weariness. I think it is worth trying to put in a wire about two-thirds as strong as that, which he will be able to wear all day without taking it out. I have in one or two other cases found springs desirable, but we do not often find them needed, and are glad to avoid them as often as possible.

*Dr. Stoddard.*—I have had several cases where it was difficult to obtain suction; one case in particular, which I reported to the Odontological Society, gave me special pleasure. I used velum rubber to line the plate with. Previously in this case I had made several plates for the patient, and had absolutely accurate impressions, but was unable to make a plate which could be retained in the mouth, and the only way the patient could use it with any comfort at all was by sifting gum tragacanth onto it every day. Finally it occurred to me to use velum rubber, and so I had a plate made and lined with two thicknesses of it. The patient has been wearing the plate with a great deal of comfort ever since, and likes it so well that she had a duplicate set made a short time ago, in case the other should become broken. I would like to ask Dr. Brackett whether he ever tried it.

*Dr. Brackett.*—I did not try that, but I should very much question if that would hold up this plate. He did have the gum tragacanth experience, if I remember rightly; and I should also question whether a smaller, weaker spring would be sufficient. I

do not understand that this gentleman takes his appliance out because it is any great burden to him; but, as a large share of his business with the outside world is transacted during the morning, in the afternoon he is quietly at home, and feels a little more the physical sense of resting without the appliance than with it.

*Dr. Wilson.*—I think one thing should be said about the velum, and that is, it deteriorates. It ought to be said, for fear somebody might be led astray. I do not question but what it was entirely successful in your case, but we all remember the velum palates made by Dr. Kingsley, and he finally discarded them.

*Dr. Stoddard.*—In answer to that question, I have lined several lower cases with velum rubber. I have one case that has been worn two years, and have seen it from time to time. The patient has kept it as clean as possible, and it shows no signs of deterioration as yet, and has no disagreeable odor. Probably in time it will deteriorate.

*President Bradley.*—There is to be a symposium on inlays, and a paper to be presented by Edward C. Briggs, M.D., D.M.D., entitled "Porcelain Inlays."

(For Dr. Brigg's paper, see page 630.)

DISCUSSION.

*President Bradley.*—The discussion of this paper is to be opened by Dr. William H. Potter.

*Dr. Potter.*—I have been asked to touch upon the history of porcelain inlays. The method first in use consisted in getting the size of the cavity by burnishing a piece of tin-foil across its aperture. The foil thus marked was trimmed to represent the size of the cavity, then pasted upon a piece of porcelain, and the porcelain cut down to the size of the tin pattern. The filling thus prepared was supposed to fit the cavity, and was cemented in place.

In the case of round, or nearly round, cavities a more accurate fit was obtained by means of circular inlay burs, which made a symmetrical cavity, into which could be fitted a cylindrical piece of porcelain corresponding in size to the bur. This proved a satisfactory method, where it did not result in a great loss of sound tooth-substance.

Dr. Herbert made glass fillings by taking an impression of a cavity in wax, and from this impression making moulds in plaster or asbestos. Into these moulds molten glass was poured. The margins of such fillings were imperfect, and the color did not hold; still they were fairly satisfactory. In 1887 Dr. Land devised the metal matrix, using gold or platinum; and from this starting-point has been developed the porcelain inlay as made and used to-day.

My own personal experience with porcelain fillings dates back to a visit with Dr. Jenkins in Dresden about two years ago. At that time I became convinced of the value of this kind of work, and have practised it ever since.

We are often asked, "How durable are porcelain fillings?" My own experience in this matter has been quite satisfactory. Dr. Körbitz, of Berlin, in a recent article, says upon this point, "A porcelain filing holds as long as it preserves the tooth. The peculiarity it has of coming out when it no longer performs its duty increases its importance as a filling-material." And he further says, "If all gold, amalgam, and cement fillings in which secondary decay exist would fall out, they would better serve the teeth than by remaining, hanging by undercuts and concealing the carious spot."

A porcelain filling shows its defects, if they exist, at once. They lie upon the surface, and are apparent. With other fillings they are too often out of sight.

*Dr. Briggs.*—I had to-day three fillings made, without any special care, just to show the different forms that they may be in. They are not perfect by any means, and I do not show them as such. I have allowed the specimens I have sent here to go as they are, to show the work as it comes from the furnace. Those fillings have not been touched at all. Of course they could be trimmed. The contour in the central incisor shows an edge at the cervical border, but that is because the enamel of the tooth has chipped away from the cervical border. It has dried and cracked off, but they are, as I say, shown to demonstrate what work can be done with the furnace in building up corners or building out contours.

*President Bradley.*—The discussion is to be continued by Dr. Stoddard.

*Dr. Stoddard.*—Mr. President, it seems to me that in the case of porcelain inlays there has been rather too much attempted, and that in a great many cases sound tooth-tissue has been sacrificed for the purpose of putting in inlays. I have put them in posterior teeth, but, as a rule, I think the application of inlays is best suited to the six anterior teeth, and that on the labial surfaces. I have seen some very extensive cases of inlays that have been made by foreign dentists, and I am satisfied that they have sacrificed not only sound tissue, but in some cases have killed the pulps of teeth, so that they might put in porcelain inlays. I think that we had better err in making too few inlays than to attempt too much and make failures of them, and bring the whole subject into disrepute. I have had a few cases where patients have said, when I suggested that they should have inlays, "My friend has had some inlays made, and they proved failures, etc.," and they will not have any in their mouths; and so a few failures may bring a very good thing into disrepute.

A very important thing in making inlays is getting a proper impression. There are a few cases where inlays are advisable, where the cavity is at the cervical margin of the tooth and extends under the gum to a greater or less extent. In these cases I think it is impossible to take a proper impression with a gold matrix, as suggested by Dr. Jenkins. In this case I think one can best get an impression by means of a pellet of gutta-percha packed into the cavity, just as in filling, except the cavity should be still moist, leaving the surface rough, and then forcing it in with a pencil of modelling compound. I have been able to take some very accurate impressions in that way.

Dr. Payne suggested a method of casting these impressions with oxyphosphate of zinc, which I have used for some time, and find very satisfactory. I case the impression with a small bit of oxide of zinc, mixed up to about the consistency of thick cream, or a little thicker, and then set it aside; and after it has hardened I set it in plaster, so that I have a plaster matrix with the cement impression in it. Then I remove the gutta-percha and make a gold matrix. The advantage of this method is that it pushes the gum back, and it is possible to get a more accurate impression than with the gold alone, and then I proceed in just the same manner that Dr. Jenkins does.

I have tried forcing the matrix in with different things, first with cotton spunk, etc., and finally with soft yellow beeswax. I have found the final method the best. I first double the gold with a ball burnisher, and then form it into the shape of the cavity as much as possible, and carry it in, and then pack in the spunk or cotton and fit it as accurately as I can to the cavity, turn over the edges, and burnish them down; then I take some soft beeswax, not too soft, just softened by the warmth of my hand, and force that in with the flat burnisher. I then take it from the cavity with an instrument, picking it out, and invest it in the asbestos, as in Dr. Jenkins's method. I place a pellet of bibulous paper over it and heat it up gradually in the furnace, the bibulous paper absorbing the wax as it softens.

I want to say a word or two about the question of high- and low-fusing bodies. That term simply means that a low-fusing body is one that will fuse in a gold matrix, without its melting, and a high-fusing body is one that will not. I have tried different kinds. It seems to be the general impression among dentists that the high-fusing bodies are the best. They are, I think, for certain things, but not necessarily for inlays. I think that I can get more accurate impression of the cavity with gold than with platinum, and consequently I prefer to use a gold matrix for that purpose, and in order to use a gold matrix I have to use a low-fusing body. The Jenkins body fuses very readily, and I have found no likelihood of its changing color or changing in any way. Of course, the low-fusing bodies are not as strong, but when I have to rely upon strength for corners, etc., I prefer to use either White's or the Consolidated Company's body, and then I know I have a very hard body which requires a high degree of heat to fuse, and I bake that in a platinum matrix, and am quite sure of the result. These other so-called high-fusing bodies I do not think are sufficiently strong for these cases. There is no danger of making a corner or tip of porcelain too strong, so I prefer to use the strongest body I can find. I think Parker's body is the strongest there is, and it is one of the best for corners. One thing which has disappointed me in porcelain inlays a great many times is that of changing color in the setting. I have time and again had a very good match in color when the inlay was completed, and then in setting spoiled it all by the cement underneath. In the case of thin inlays I think it is unavoidable. The porcelain is more or less translucent: if you put an opaque substance under it, it will show through. Another thing, if the inlay is seen from different points of view, or in different lights, the effect is not so good as you expect, and therefore to that extent porcelain is disappointing, although it is much preferable, of course, to any other material we have, from an æsthetic stand-point.

In regard to the wearing qualities of inlays,—that is, the question of preserving the teeth, the tendency of the cement to dissolve, etc.,—I think that is a question not to be considered very much. Porcelain has proved to be a very durable filling-material. Where the line of cement is comparatively narrow, which we all hope to have in making an inlay, the cement shows but very little tendency to dissolve. After it has dissolved to a certain depth, it is self-protecting, to a certain extent. I have seen inlays which have been in a great many years where I could feel around the margin of the cavity with a very fine explorer, and yet which seemed to be preserving the teeth very well, and were likely to do it for many years longer. That point which Dr. Potter brought out seems to me a very good one. If a porcelain filling comes out, you know it at once. It may start to decay a little at the margin, but just the minute the decay goes under the filling, then we know it is time to put in another one.

I do not think of anything else to offer on this subject, except that where one can use to good advantage the round inlays which we spoke of one or two meetings ago, they are very much preferable to the inlays made by the Jenkins method, so-called, or any of these methods where it is necessary to have a matrix.

*President Bradley.*—The committee informs me that Dr. Allen was to participate in the discussion, but he wished me to present his excuses to the Academy, saying that he felt he must leave in order to reach his home this evening.

Dr. Andrews said he should speak principally of the inlays in the teeth that are in the museum at Cambridge. I think he has spoken to the Fellows of the Academy about them upon some other occasion, and he was going to speak of them at this time, saying that if the people who made those inlays could do so well with the imperfect instruments which they had to work with, of course we should do still better work with the appliances we have now.

This evening we have with us several visitors, and the courtesies of the floor will be extended to all who are present with us. Among them is Dr. Patten, a guest of Dr. Potter. I think he is interested in inlays, and we should be very glad to hear from him.

*Dr. Patten.*—I feel, after what has been already said, that my limited experience and what little I have to say will not be of very much profit. Most of the remarks have been along the lines of low-fusing bodies, and the experience has been in that line of work. My own has been confined almost entirely to high fusing. This last winter I have done some slight work with the Jenkins body. I must say that there are a great many things about working with a gold matrix that appealed to me very strongly, especially in cavities in the labial surfaces of teeth more particularly than in any other location.

Dr. Potter brought up a feature of porcelain work that has struck me forcibly, and that is the impression it has made on the public. So far as I have been able to learn, it has been received with a great deal of enthusiasm by patients, and I presume that the doing away with large display of gold is the particular feature that has appealed to them. It has been my experience that wherever I have been able to replace gold fillings with suitable porcelain fillings, well matched and well made, my patients have been especially pleased and gratified, and have felt the need of more of it. Another feature that has recommended it very strongly to me is the use that I can make of it in teeth that you might term frail, where the application of gold by the old-time methods would be out of the question. In fact, they would be barred out because the teeth would not be of sufficient strength to stand the application of a large gold filling. In that case porcelain is pre-eminently the finest thing to use, because of the fact that pressure is not required, and we get the sustaining strength of the cement and also the support of a large body of porcelain. In these cases I think all those who have followed this line of work will bear me out in saying that it is the filling to use.

There are, of course, in everybody's experience many dismal failures to be recorded and to stare us in the face, but the element of permanency or the lack of permanency is one that we can almost abstain from discussing. If any of you happen to have read the article in the *Items of Interest* for January, by Dr. Capon, of Philadelphia, relating his twelve years' experience, it seems to me his experience would be satisfactory to almost anybody in the line of permanency; and most of you gentlemen, I presume, who have seen his work, must admit that the results he obtained are entirely satisfactory. His only "failure," I think he calls it, was a tip on a central, and he said that it had been in place nine years before it required attention. I happened to be in his office just about a year ago, when he was replacing that tip on the central, and he told me that was put on nine years before, and he expected the one he put on that day would stand fully as long. That is about as much as we can expect of any kind of a filling, and without considering the decided advantages of the appearance.

I have been strongly tempted many times to throw aside the platinum matrix, because of inability on my part (I think it is merely a matter of lack of skill, perhaps, in manipulation, which may come after more experience), and adopt the gold matrix, but several things have deterred me from doing this; and in my experience I would hardly recommend anybody who has adopted a certain method to throw it aside for another. If a man has adopted the low-fusing bodies with gold matrices, his best plan is to follow it out, as both the high- and low-fusing bodies have their special recommendations; but I am satisfied, so far as I have gone, that what failures I have had, and they have been many, have been the result not of the method I am using, but of my lack of manipulation or skill, which I hope in time to overcome.

In regard to shades, the outfit of Dr. Jenkins presents some very natural and desirable shades, but I have found in the last few months that my early methods of matching the porcelain of the teeth have been very defective, because of the fact that I have endeavored to match the teeth by securing shades of porcelain as a result of mixing one or more in the start, which I have found to be a great mistake. At the present time I adopt a little different method, and, if I might so express it, there is a very great desirability in being able to see the underlying shade.

There is one special feature I would like to mention in the preparation of the cavity, and that is, the result largely depends upon the margins. They should be sharp, clearly defined, and very accurate, and, of course, Dr. Jenkins's method of subjecting them to considerable polish is a very admirable one; but I fail to see wherein he can apply that high polish to the entire margin of the cavity in all cases. In many cases you can, and in a great many cases you cannot; but it is desirable to have the margins absolutely true and perfect. They do not need to be parallel, but should be square and sharp, in order that the matrix may be clearly defined, and therein lies the secret of bringing out the porcelain in perfect shape.

I was interested in the remarks made by those who preceded me, in regard to the setting of porcelain by cement. My early experience prompted me to do as I had been advised by others, protect the porcelain and the cement with the rubber dam, but my later experience has been quite widely different; and if the porcelain comes out with the margin a little too sharp and perhaps a little too high, I do my trimming before setting the porcelain, and after the porcelain is set with cement I simply wipe the cement off and let it go. As Dr. Potter put it, cement will set harder in saliva than if kept dry; therefore I simply do away with that trouble.

Dr. Potter has called my attention to that question about the shades. I start now with a shade which I seem to see underlying the enamel, and follow that up with another which will approximate, and finally finish the whole with a shade which is as nearly a perfect match to the enamel as it is possible to get it. In ninety-nine cases out of a hundred the shade that I start with will be the lightest, because I think in the majority of cases the dentine is apt to be lighter than the enamel, and the enamel will perhaps be of a blue, green, or yellow shade. In that way I get a piece of porcelain that will more closely resemble the tooth than in any other way.

*Dr. Belyea.*—I would like to inquire if Dr. Stoddard has made any other experiments with the Canada balsam?

*Dr. Stoddard.*—No, I have not. I have not dared trust it in the mouth, but I set one three or four months ago, and I still look at it from time to time, and cannot see that any change has taken place, so I think theoretically it is all right; practically I should hardly dare trust it in the mouth.

*Dr. Meriam.*—I have heard of specimens of Japanese lacquer that have been in sea-water for years and came out uninjured. I think that some of our manufacturers have, whether they do now or not, offered a lacquer that resisted hot alcohol, and very likely along some of these lines we would find a cement that would suit us. I never hear this question discussed but that I am surprised at our lack of patriotism in not putting on record that the matrix was first made here in Boston earlier than 1875. It seems extraordinary to me that it should be so.

*Dr. Decker.*—Among many other valuable points I have gathered from Dr. Clapp, one applicable to this discussion is the manner of mixing the cement to preserve its original color. It is done with a wooden spatula. The metal spatulas unfortunately discolor it by wearing off. I simply take an orange-wood stick, about one-quarter of an inch in diameter, and whittle one end, then file it perfectly smooth to about one-sixteenth of an inch in thickness. This can be dipped in a liquid with immunity, and is strong enough to apply the force. After it has been used a short time, it becomes coated with cement and ceases to absorb the liquid. After mixing I scrape off the wood with a steel spatula. When it becomes too thick it is filed down again, and makes an admirable instrument. It obviates the discoloration of the cement, because there is no oxidization of the wood.

In regard to leaving a layer of cement over the inlay to preserve it from moisture, it seems to me impracticable, as cement left on the smooth slab and moistened, within a few minutes after it has been mixed will by capillary attraction become loosened and wet between the cement and the slab, and it seems to me that the same capillary attraction of the saliva would run immediately under the cement on the smooth surface of the tooth and the inlay, and moisten the cement inside almost as quickly.

*Dr. Briggs.*—I would like, for the encouragement of those who do not know how well these stand, to speak of one case that comes to my mind of a corner that I put on a front tooth, about the size of the corner of that tooth that was passed around.

A young man bit off a corner, and I put one on a little over twenty years ago, and he wore it eight or nine years, and then had an accident and broke the porcelain right in two. He might have broken his tooth. I put on another one, and that one he is wearing to-day, and his tooth is in perfect condition. That is one only of a great many cases.

My experience is not confined only to the Jenkins porcelain. I have put them in for over twenty years, but of course the old method was very laborious, and I did not seek to do anything except in very conspicuous places; but with the Jenkins method I put it in all parts of the mouth, and not for the purpose, as was intimated by Dr. Stoddard, of getting the porcelain in at any cost, but put it in teeth where it would not admit of anything else, unless you put in cement, or destroyed the pulp in order to build up a gold filling; and it is in that character of teeth that I have found it very helpful, in just the cases where our best gold operators would decline to attempt to put in a gold filling under the conditions that existed.

*Dr. Cook.*—I would like to speak of the impression that I gathered from Dr. Potter, which was that Dr. Pond in his experiments found that cement when wet was just as strong as when dry. I got the other impression,—*i.e.,* that if the cement is covered over with wax or resin or paraffin, it does not last much better than if allowed to be wet at once, but if kept dry for a long time, that was the cement that stood the high pressure.

*Dr. Belyea.*—That was what I also gathered from Dr. Pond's remarks.

*Dr. Ainsworth.*—I should like to know if there is any choice in furnaces for low- or high-fusing bodies?

*Dr. Briggs.*—The Jenkins, of course, is good, but I have used lately the Ash or the Hammond. The Ash is a very simple furnace, and is an electric furnace. It is made about the size for an inlay. The Hammond furnace is more elaborate, and that may be used for all bodies. I have often used the Parker body in that, although it has burned out one or two muffles.

*Dr. Stoddard.*—I still stick to the gas-furnace,—for very high-fusing bodies, the Parker furnace, but for the Jenkins inlay and the low-fusing bodies I use the Ash furnace.

On motion, adjourned.

CHARLES H. TAFT, D.M.D.,
*Editor American Academy of Dental Science.*

